# Monitoring Physical Activity in Students with Intellectual Disabilities: The Contribution of Physical Education, Gender and Disability Level

**DOI:** 10.3390/s26061808

**Published:** 2026-03-13

**Authors:** Yannis Ntovolis, Lida Skoufa, Christina Evangelinou, Vassilis Barkoukis

**Affiliations:** 1 Department of Physical Education and Sport Science, Aristotle University of Thessaloniki, 57001 Thessaloniki, Greece; ntovolis_io@outlook.com (Y.N.); lida.skoufa@yahoo.gr (L.S.); 2 Department of Physical Education and Sport Science, Aristotle University of Thessaloniki, 62500 Serres, Greece; evaggeli@phed-sr.auth.gr; 3 Department of Life and Health Sciences, Frederick University, Nicosia 1036, Cyprus

**Keywords:** intellectual disabilities, physical education, pedometer

## Abstract

Individuals with intellectual disabilities (IDs) consistently demonstrate lower levels of objectively measured physical activity (PA) compared to the general population, yet limited evidence exists regarding how activity accumulated during physical education (PE) contributes to overall daily movement within structured school contexts. Within the school setting, PE represents one of the primary structured opportunities for engaging students with IDs in PA. Although objective physical activity monitoring approaches are recommended for school-based PA assessment, limited evidence exists on the contribution of PE to total school-day activity in students with intellectual disabilities, a gap addressed in the present study. In this context, the present study objectively recorded PA levels of students with IDs both during PE lessons and across five school days, in order to examine the contribution of PE to overall PA. Potential differences in PA according to gender and severity of the ID were also examined. Twenty students aged 15–25 years with mild and moderate IDs participated in the study. PA was assessed using the YAMAX Power Walker EX-510 pedometer, which automatically recorded step counts. The results indicated that only six participants reached step-count reference values. Students with mild IDs accumulated significantly more steps than those with moderate IDs, while male students were more physically active than female students, both during PE lessons and across the school day. PE lessons contributed approximately 4% to the total PA accumulated across the five monitored school days. These findings highlight the limited contribution of PE to overall PA and underscore the importance of promoting greater movement opportunities within adapted PE lessons.

## 1. Introduction

Physical inactivity and prolonged sedentary behavior have emerged as major global public health concerns. A substantial proportion of the global population fails to achieve recommended levels of physical activity (PA), contributing to increased risks of cardiovascular disease, metabolic disorders, certain cancers, and premature mortality [1,2]. In addition, growing evidence indicates that sedentary lifestyles are associated with adverse mental health outcomes and reduced overall well-being [3]. Within this broader public health context, understanding and promoting PA as a protective health behavior becomes particularly important. PA is a key determinant of physical and mental health across the lifespan [1,4,5,6]. Despite its well-established benefits, individuals with intellectual disabilities (IDs) consistently demonstrate lower levels of PA and higher levels of sedentary behavior compared to the general population [7,8,9]. This persistent inactivity highlights the need for structured and context-specific opportunities that can effectively support PA engagement in this population.

Schools constitute one of the most structured and accessible environments for promoting PA among children and adolescents with IDs. Although PA may also occur in community and family settings, participation outside school is often influenced by environmental and social barriers, including limited access to inclusive programs and reliance on caregiver support [10,11]. In addition, organizational constraints such as limited staffing, transport difficulties, inconsistent routines, and insufficient structured support have been identified as significant barriers to sustained engagement in PA among individuals with IDs [12]. Caregiver knowledge, beliefs, and competing responsibilities may further influence opportunities for regular participation [13], while low motivation, health-related limitations, and predominantly sedentary daily routines can also restrict consistent PA involvement [12]. As a mandatory component of the school curriculum, physical education (PE) provides an institutionalized and supervised context for engagement in organized PA under the guidance of trained teachers [14,15]. Participation in structured PE programs has been associated with improvements in physical fitness, motor competence, and psychosocial outcomes in students with IDs [16,17,18]. Given this central role, examining the extent to which PE contributes to students’ overall daily PA is of particular importance. However, existing studies have not specifically quantified the proportional contribution of PE to total accumulated daily PA among students with IDs. Quantifying this contribution is essential for informing PE design and optimizing school-based PA promotion strategies within special education settings.

To address this need for objective monitoring, the present study aimed to record PA levels of students with IDs during PE lessons and across the school day, and to assess the contribution of PE to total accumulated PA. In addition, differences in PA according to gender and severity of IDs were examined. It was hypothesized that: (a) step counts accumulated during PE lessons would significantly predict total accumulated school-day step counts; (b) males would demonstrate higher PA levels than females during PE and across the school day; and (c) students with mild IDs would demonstrate higher PA levels than those with moderate IDs.

Despite the recognized importance of PE, the existing literature lacks comprehensive objective evidence regarding the amount of PA accumulated specifically during PE lessons and the extent to which PE contributes to overall daily activity. This gap limits the development of evidence-based PE design and targeted PA promotion strategies within special education settings.

The remainder of this article is organized as follows: Section 2 presents the Related Works, Section 3 presents the Materials and Methods, Section 4 presents the Results, Section 5 presents the Discussion, Section 6 presents the Limitations and Section 7 presents the Conclusions.

## 2. Related Works

### 2.1. PA Levels in Individuals with IDs

An ID is a developmental condition characterized by significant limitations in intellectual functioning and adaptive behavior, including conceptual, social, and practical skills necessary for independent living [19]. In high-income countries, approximately 2–3% of children are estimated to have IDs, while prevalence rates may be higher in middle-income settings [20]. An ID is commonly classified according to severity (mild, moderate, severe, or profound), as well as by etiology (genetic and non-genetic causes). A substantial body of evidence indicates that individuals with IDs engage in lower levels of PA compared to the general population [7,8,9]. For example, only a small proportion of adults with IDs meet the recommended minimum levels of moderate-to-vigorous PA (MVPA) per week [18], with participation rates being markedly lower than those observed in the general population [8]. Systematic reviews further highlight that insufficient PA in individuals with IDs is associated with increased sedentary behavior and elevated health risks [16,17,21].

In addition to reduced activity levels, individuals with IDs are at greater risk of inactivity-related health conditions, including obesity, cardiovascular disease, and multi-morbidity [22,23,24,25,26]. Nevertheless, structured PA interventions of sufficient intensity and frequency have been shown to improve physical fitness, mental health, and quality of life in this population [16,17]. These findings underscore the importance of identifying effective contexts for promoting sustained PA engagement.

### 2.2. Gender Differences in PA Among Individuals with IDs

Previous research has consistently reported gender differences in PA participation among individuals with IDs. A systematic review and meta-analysis demonstrated that females with IDs are significantly less physically active than males, particularly in terms of daily step counts and MVPA [27]. More recent evidence suggests that females with IDs may encounter additional structural and psychosocial barriers to PA participation, including reduced access to organized programs and lower perceived competence [28]. Gender disparities in PA have been linked to broader inequalities in health and well-being among individuals with IDs [29]. Importantly, some studies suggest that gender differences may vary depending on context, with smaller or less consistent differences observed during structured activities such as PE compared to unstructured school periods [30]. These findings highlight the importance of examining gender when monitoring PA in school-based settings.

### 2.3. Severity of IDs and PA Levels

The severity of an ID has also been identified as a determinant of PA participation. Evidence indicates that individuals with moderate IDs tend to engage in lower levels of PA compared to those with mild IDs, both during childhood and adulthood [19,29,30,31]. Objective assessments using accelerometry and pedometry have shown that greater severity of an ID is associated with lower daily step counts and reduced likelihood of meeting PA recommendations [7,32,33]. Although individuals with moderate IDs generally demonstrate lower PA levels, variability within severity groups suggests that contextual and modifiable factors, such as environmental support, structured opportunities, and motor skill development, play a critical role in shaping activity behavior [33]. These findings emphasize the importance of examining PA patterns across severity levels within structured educational environments. Importantly, although the above-mentioned literature reports an association between ID severity and PA levels, this should not be interpreted as evidence of reverse causality. Accordingly, the present study investigates the association between ID severity and PA levels rather than any possible causal links.

### 2.4. Monitoring PA in Educational Contexts

Given the central role of the educational and social environment in the daily lives of individuals with IDs, professionals and caregivers may significantly influence PA behavior [12,13,34]. Within school settings, PE teachers represent key agents who can shape students’ engagement in structured PA. For many students with IDs, PE constitutes one of the primary structured contexts in which regular PA participation occurs. Objective PA monitoring has been applied in school settings to examine activity patterns of children with disabilities across different segments of the school day. Accelerometer-based studies have quantified PA and sedentary time during physical education (PE), recess, and lunchtime, enabling context-specific comparisons within the school environment [35]. In parallel, pedometry has been used to assess step-based activity volumes in individuals with IDs, examining variations across school days, non-school periods, and days with versus without adapted physical education (APE), as well as across levels of ID severity [36]. Methodological research has also addressed reliability considerations in pedometer monitoring within ID populations [37], while evidence indicates potential step underestimation during dynamic APE movements [38]. Wearable-based PA monitoring in individuals with IDs has been widely implemented using accelerometer-based field protocols. Systematic reviews have identified substantial variability in methodological decisions, including monitor selection, placement, wear-time criteria, and cut-point application, highlighting the lack of standardized accelerometer protocols in this population [39,40]. School-based investigations have further applied objective accelerometer measures to distinguish activity accumulated during school and after-school periods, emphasizing the importance of contextual segmentation in understanding PA behavior among youth with IDs [41].

Despite the growing body of wearable-based monitoring research, previous studies have not simultaneously quantified the proportional contribution of PE to total accumulated school-day PA while also examining gender and severity differences within a unified analytical design. This methodological gap is addressed in the present study.

## 3. Materials and Methods

To provide a structured overview of the study design, the monitoring framework consisted of three main stages: (a) objective recording of step counts using pedometry during both PE lessons and habitual school days, (b) aggregation of step-based indicators for PE-specific and total monitored activity, and (c) statistical examination of the contribution of PE to overall accumulated activity and of differences according to gender and ID severity.

### 3.1. Participants

Twenty high school students (*N* = 20) attending a Special Education School in southern Greece participated in the present study. Participants were aged between 15 and 25 years (M = 19.4, SD = 2.72), and 14 were male. In the Greek special education system, students with IDs may remain enrolled in upper secondary special education schools beyond the typical age range of general education; therefore, the observed age range reflects the institutional structure of the national educational framework. Four participants had a moderate level of IDs, while 16 had a mild level. A convenience sampling method was used through contacts of the authors with regional educational authorities. Inclusion criteria were a diagnosis of an ID, absence of motor impairments, no current medication, and no comorbid conditions. The level of the ID had been previously assessed by a psychologist at the educational authority’s official diagnosis center using the Wechsler Intelligence Scale for Children—Third Edition (WISC-III).

Prior to the commencement of the study, parents and guardians were informed about the purpose and significance of the research and provided written consent for their child’s participation. Although the sample included participants above 18 years of age, all were enrolled in a Special Education Secondary School and were under legal guardianship in accordance with the national special education framework. The study procedures were explained to all participants in an accessible language, and their willingness to participate was verbally confirmed prior to data collection; participation did not proceed in cases of expressed unwillingness.

### 3.2. Experimental Setup

PA was assessed using the YAMAX Power Walker EX-510 pedometer (Yamax Corporation, Tokyo, Japan), which features a highly accurate triaxial motion sensor (3D), a dual-readable 6-bit crystal display, a three-dimensional accelerometer, a 30-day memory, a 30-week total memory, a 24 h clock, and a safety strap. The YAMAX Power Walker EX-510 operates as a closed-system pedometer providing aggregated step-count outputs via its internal algorithm. Raw acceleration signals are not accessible for external processing. Therefore, no additional signal filtering or machine learning-based processing was performed. The study relied on the device’s validated step-count output as provided by the manufacturer. The device provides a measurement accuracy of approximately 98.5% and has a one-year battery life. Previous studies have supported the reliability and validity of the Power Walker EX-510 [42,43], and earlier YAMAX models have also demonstrated high consistency when compared with other objective PA measurement tools [44].

The pedometer weighs 24 g (including the built-in battery), and its dimensions are 76 mm × 33.5 mm × 10 mm. It measures step count, calories, fat burned, distance, and active time, and operates within a temperature range of 0–40 °C. Step count accuracy is approximately 3%. The device includes a non-step filter, meaning that if fewer than 11 steps occur followed by a pause of at least five seconds, those steps are not recorded, thereby increasing measurement precision. Steps were used as an objective indicator of PA. Objectivity was ensured through the use of a validated triaxial pedometer that automatically records step counts based on acceleration signals, without reliance on self-report or observer-based estimation. The pedometer was placed at the waist using an elastic band and secured inside a foam case, positioned centrally and slightly to the right side of the body. The placement was selected to ensure both measurement consistency and participant safety and was standardized across all participants.

### 3.3. Experimental Protocol

An initial meeting was held with parents and guardians to explain the operation and requirements of the pedometers throughout the study. A demonstration on how to set up and use the pedometer was provided, followed by detailed instructions and a briefing on research procedures, anonymity, and voluntary participation. Parents were informed that participation could be withdrawn at any time without consequences.

After obtaining consent from parents/guardians and institutional permission from the school administration, pedometers were distributed to the parents for use by the students. To ensure familiarity and accuracy, the pedometers were tested for one hour during a PE lesson prior to the official measurement period.

In order to record the participants’ physical profile, height and weight were measured under standardized conditions (light clothing and no shoes) in an appropriate setting [45]. The study measured both the number of steps taken during PE lessons and the total number of daily steps. Students wore the pedometers throughout the day, except between 15:00 and 17:00 (resting period) and during nighttime sleep. Parents were instructed to attach the pedometer each morning at 08:00 and remove it at bedtime. They were also asked to remove the device during bathing or swimming. This procedure was followed for five consecutive school days. All participants attended PE lessons twice per week, ensuring consistency in the measurement period.

### 3.4. Demographics and Content of the PE

Demographic information, including gender, age, level of ID, height, and weight, was recorded (Table 1). To facilitate the interpretation of findings, a diary was maintained by the school’s physical educators, documenting the content of each PE lesson. PE is a mandatory curriculum-based school subject focused on developing physical fitness, motor skills, teamwork, and healthy lifestyle habits through structured physical activities and sports. The PE lessons followed the National Curriculum [46] and included team games (e.g., basketball and football), individual sports (e.g., athletics), and other activities, such as traditional dances, aerobic exercises, dynamic balance, and orientation tasks. The course was delivered twice per week, with each session lasting 45 min and including a warm-up phase, a main activity phase, and a short cool-down period. All sessions were conducted during morning school hours (08:30–13:30) and were uniform across classes in both structure and intensity.

### 3.5. Data Analysis

Statistical analyses were conducted using SPSS version 24. The assumption of normality was evaluated using the Shapiro–Wilk test (significance level of α = 0.05). One study variable (i.e., mean step counts during PE lessons) demonstrated a significant deviation from normality. However, inspection of skewness and kurtosis values (scores < 1.5) and visual inspection of Q-Q plots indicated no substantial departure from normality, and given the robustness of parametric tests to moderate violations of this assumption, parametric analyses were performed. Both descriptive and inferential statistics were employed. Independent-samples t-tests were performed to examine gender differences in PA levels and differences in mean step counts according to the severity of the ID. In addition, a regression analysis was conducted to evaluate the contribution of PE lessons to overall PA.

## 4. Results

### 4.1. Contribution of PE to Overall PA in Individuals with IDs

A linear regression analysis was conducted to examine the contribution of PE to overall PA levels. Total step counts accumulated across the five monitored school days were entered as the dependent variable, while step counts accumulated during PE lessons were entered as the independent variable. The regression model was not statistically significant, F(1, 18) = 0.96, *p* > 0.05, and explained a very small proportion of variance in total step counts (Adjusted R^2^ = −0.002), indicating that PA accumulated during PE lessons did not predict overall PA levels. Descriptive analyses, comparing steps accumulated during PE lessons with total step counts across the five monitored school days, showed that PE lessons accounted for approximately 4% of participants’ total PA accumulated across the five monitored school days.

### 4.2. Gender Differences in Mean Steps

**Figure 1:**
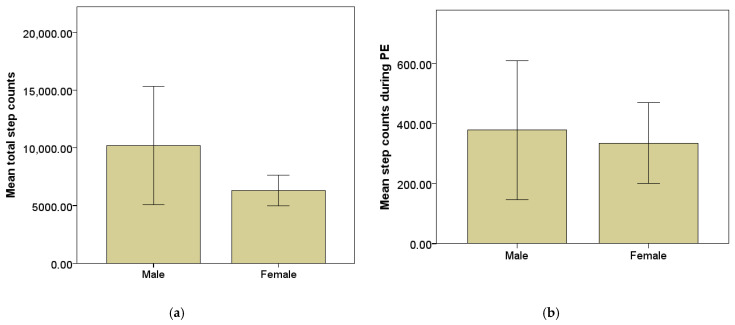
Mean step counts accumulated across the five monitored school days (±SD) by gender for (**a**) total step counts and (**b**) step counts during PE lessons.

Independent-samples t-tests revealed significant gender differences in PA levels. Male participants accumulated significantly more total step counts across the five monitored school days than female participants, t(16.29) = 2.64, *p* = 0.018. Similarly, males recorded significantly higher step counts during PE lessons compared to females, t(16.29) = 2.63, *p* = 0.018. These differences are illustrated in Figure 1a,b.

### 4.3. Differences in PA by Severity of ID

**Figure 2:**
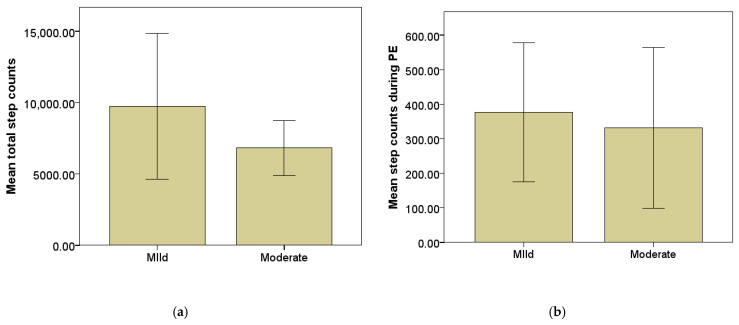
Mean step counts accumulated across the five monitored school days (±SD) according to the level of IDs for (**a**) total step counts and (**b**) step counts during PE lessons.

Independent-samples t-tests revealed significant differences in PA levels according to ID severity. Male participants accumulated significantly more total step counts across the five monitored school days than female participants, t(16.29) = 2.64, *p* = 0.018. Similarly, males recorded significantly higher step counts during PE lessons compared to females, t(16.29) = 2.63, *p* = 0.018. These differences are illustrated in Figure 2a,b.

## 5. Discussion

The purpose of the present study was to record and examine the contribution of PE lessons to the overall PA of students with IDs. Furthermore, differences in PA between genders and degrees of severity of IDs were investigated. The number of steps was used as an indicator of students’ PA, as steps provide an objective parameter reflecting the time spent participating in PA [47].

The findings indicated that participants’ step counts were lower than step-based reference values commonly reported for adolescents in the general population [48], while being closer to those reported for adults [47]. With respect to the PA levels of the study’s participants, it should be noted that although they did not reach the recommended PA levels for adolescents, their mean step counts were higher than those reported in earlier studies with people with IDs [49,50] but still lower than those of the general population [51]. These findings highlight the persistent problem of inadequate PA among individuals with IDs and underline the need to address this issue, as insufficient activity is associated with multiple adverse health outcomes [52]. Recent systematic reviews have also confirmed that programs with higher frequency and sustained duration (≥3 sessions per week) lead to significant improvements in fitness, health, and well-being among individuals with IDs [10,11]. In Greece, the two PE sessions per week may limit sustained engagement in PA. Evidence from systematic reviews suggests that higher-frequency programs (≥3 sessions per week) are associated with improved health and fitness outcomes among individuals with IDs [16,17].

Importantly, one of the main findings of this study was that PE contributed only minimally to students’ total PA. These findings are consistent with prior research demonstrating limited physical engagement during PE among students with IDs. Previous research has shown that only a small proportion of PE lesson time is spent in moderate-to-vigorous activity and that participation largely depends on instructional adaptations and teacher support [18]. Together with the present results, this evidence suggests that conventional PE formats may be insufficient to substantially increase overall accumulated PA among students with IDs.

Several factors may explain this limited contribution. Research in mainstream settings indicates that less than half of PE time is spent in actual movement [53], and this proportion is likely even smaller for students with IDs, who often require individualized instruction and additional time to process directions. Bertills and Björk [54] also noted that PE teachers frequently face practical barriers, such as limited resources, large class sizes, and a lack of specialized training in adapted methods when teaching students with diverse disabilities. Beyond these structural limitations, broader school-level and systemic factors, such as insufficient teacher support, inadequate facilities, and limited inclusion-oriented school policies, also constrain participation for children and adolescents with disabilities [55]. In addition, the lack of a specific adapted physical education curriculum, as well as the heterogeneity of the participants’ needs, is considered a barrier to successful physical education implementation within schools. Taken together, these findings indicate that, as currently implemented, PE makes only a modest contribution to students’ overall PA. Enhancing its effectiveness requires coordinated action at the curriculum and policy levels to ensure that PE provides more consistent and meaningful opportunities for physical engagement.

In support of our second and third hypotheses, our findings showed statistically significant differences between males and females, and between students with mild and moderate IDs, both during PE and across the monitored school week. These hypotheses were formulated to examine whether students with different individual characteristics demonstrate different PA patterns. Our results confirmed that males and students with mild IDs were more active compared to females and students with moderate IDs, a finding consistent with prior evidence [30,56]. Possible explanations include differences in perceived competence, motivation, or available opportunities, as well as social stereotypes that may discourage female participation in active or competitive activities [10,57]. These findings suggest that females and students with moderate IDs are at higher risk for the adverse effects of inactivity. Policymakers and physical educators should be aware of these disparities and design targeted interventions that foster equal participation. For instance, physical educators should pay specific attention to females’ participation in the lesson. Likewise, policymakers should ensure that curricula include accessible activities suited to varying ability levels.

## 6. Limitations

This study has several limitations. Steps were used as the sole indicator of PA, which does not capture intensity or type of movement. Although step counts are a valid proxy for overall activity, accelerometer-based measurements could provide a more detailed assessment of PA intensity and sedentary time [56]. The small sample size and focus on a single educational setting also limit the generalizability of the findings. Future research should use multiple objective measures, involve larger and more diverse samples, and examine multilevel interventions that integrate individual, social, and environmental factors. In addition, the use of a closed-system pedometer provides aggregated step counts without access to raw acceleration data. Therefore, advanced signal-processing or CNN-based feature extraction approaches applied directly to raw inertial signals could not be implemented. Future studies employing open-access wearable sensors may allow further optimization through deep learning-based signal enhancement. Despite these limitations, the study provides empirical evidence regarding the contribution of PE to accumulated step counts in students with IDs within a school setting. The findings indicate that PE accounts for a relatively small proportion of overall monitored activity and that gender and ID severity are associated with differences in step counts.

## 7. Conclusions

The findings of this study underscore the urgent need to enhance PA opportunities for students with IDs within the school environment. As PE remains one of the few structured and inclusive contexts available to this population, its redesign is critical. Expanding lesson duration and frequency, integrating adapted and cooperative activities, and providing teacher training in inclusive pedagogy could help bridge the current activity gap. Policymakers and educators should collaborate to ensure that PE in special education not only promotes movement but also fosters health, confidence, and social inclusion for all students. In addition, concerted efforts should be made to promote PA in females and people with moderate IDs. In this respect, behavior change guidelines should be developed both within and outside schools to engage females and people with moderate IDs. Similarly, fitness programs tailored to these populations should be developed to accommodate their needs. Overall, a more concerted and focused effort should be made by policymakers to promote PA in people with IDs.

## Figures and Tables

**Table 1:** Descriptive statistics of the sample and tested variables.

		Mild		Moderate		Total	
Variable	Gender	Mean	SD	Mean	SD	Mean	SD
*N*	M	12	–	2	–	14	–
	F	4	–	2	–	6	–
Age (years)	M	19.92	2.57	17.50	3.54	19.57	2.71
	F	19.50	3.32	18.00	2.83	19.00	2.97
BMI (kg/m^2^)	M	23.60	6.26	22.11	0.55	23.39	5.78
	F	29.20	3.66	23.63	5.76	27.34	4.79
PA (steps/day)	M	10,994	5083.18	5341	1695.24	10,186	5128
	F	5945	1438.09	7024	928.23	6305	1313
PE (steps/day)	M	411	234.96	184	28.07	378	231
	F	365	160.41	275	52.11	335	135

## Data Availability

The data presented in this study are not publicly available due to privacy and ethical restrictions related to participant confidentiality. Data may be made available upon request from the corresponding author.

## Abbreviations

The following abbreviations are used in this manuscript:

PA	Physical activity
IDs	Intellectual disabilities
PE	Physical education
